# Raeder's Syndrome: An Uncommon Cause of Partial Horner Syndrome Secondary to Petromastoid Inflammation

**DOI:** 10.7759/cureus.86269

**Published:** 2025-06-18

**Authors:** Yin Min Htwe, Vinod Warrier, Shahid Nasim, Sathyanarayana Gowda

**Affiliations:** 1 Acute Internal Medicine, Southend University Hospital, Southend-on-Sea, GBR; 2 Internal Medicine, Mid and South Essex NHS Foundation Trust, Southend-on-Sea, GBR; 3 Acute Medicine, Southend University Hospital, Mid and South Essex NHS Foundation Trust, Southend-on-Sea, GBR

**Keywords:** botulinum injection, gradenigo syndrome, neuropathic pain management, occulosympathatic pathway, painful partial horner syndrome, petrous apicitis, raeder's syndrome, therapeutic benefit, trigeminal nerve

## Abstract

We report the case of a 58-year-old woman who initially presented with left-sided headache, facial discomfort, and a partial Horner syndrome affecting her left eye. Imaging revealed inflammatory changes within the petromastoid air cells, consistent with petrous apicitis. The clinical and radiological features were aligned with Raeder's syndrome, the rare cause of painful partial Horner syndrome.

This case highlights the importance of early recognition of this uncommon but treatable medical condition.

## Introduction

Raeder paratrigeminal syndrome, also known as Raeder's syndrome or paratrigeminal neuralgia, is a rare postganglionic variant of Horner syndrome. It is characterized by severe, unilateral facial pain and headache in the distribution of the ophthalmic division of the trigeminal nerve, accompanied by ipsilateral ptosis and miosis with preserved facial sweating [1]. The preservation of facial sweating is a key distinguishing feature from classic Horner syndrome, which includes ipsilateral ptosis, miosis, and anhidrosis, and reflects a lesion in the paratrigeminal (post-post-postganglionic) portion of the oculosympathetic pathway [2].

Raeder’s syndrome is most commonly associated with lesions in the petrous apex of the temporal bone, such as petrous apicitis. The petrous apex, a part of the temporal bone located near the base of the skull, is a region where inflammation or compression can affect multiple cranial nerves and sympathetic fibers. Despite its significance, pathology in this area is rarely encountered in routine clinical practice, contributing to the underrecognition of the syndrome. 

The clinical significance of Raeder's syndrome lies in its subtle but important diagnostic implications. It is often presented with features that mimic other causes of painful Horner syndrome, such as carotid artery dissection, cavernous sinus lesion, or internal carotid artery aneurysm. Hence, early recognition of this syndrome and an understanding of its anatomical basis can help clinicians narrow the differential diagnosis, guide appropriate imaging, and facilitate treatment.

Due to its rarity and overlap with more common neurological or vascular conditions, Raeder's syndrome is often underdiagnosed. Fewer than a dozen well-documented cases have been reported in the literature, and their pathophysiology remains an area of ongoing clinical interest. Heightened clinical awareness is essential, particularly in patients presenting with unilateral facial pain, partial Horner syndrome, and no apparent cause on initial examination. 

## Case presentation

A 58-year-old woman presented to the same-day emergency care (SDEC) with a five-day history of left-sided headache and facial pain. She described the pain as dull and persistent, localized around the left temporal region and periorbital area, radiating down to the left cheek occasionally, and not aggravated by touch or changing in position, and no special time of the day. Three days later, she started noticing drooping of the left eyelid. However, she denied any visual changes such as diplopia or blurred vision and tearing of the eyes. She did not have any infectious or coryzal symptoms at the time of presentation.

Approximately five weeks prior, she had experienced left ear blockage, for which she was prescribed a seven-day course of oral antibiotics for presumed sinusitis by her general practitioner. Her symptoms were improved at that time upon review, and there was no history of fever, ear discharge, hearing loss, tinnitus, or vertigo. She denied any preceding trauma or injury to the head and neck.

Her past medical history was notable for spasmodic torticollis, managed with regular botulinum toxin injections every six months under neurology follow-up. She was not on anticoagulation, had no vascular risk factors, and was a non-smoker.

**Figure 1:**
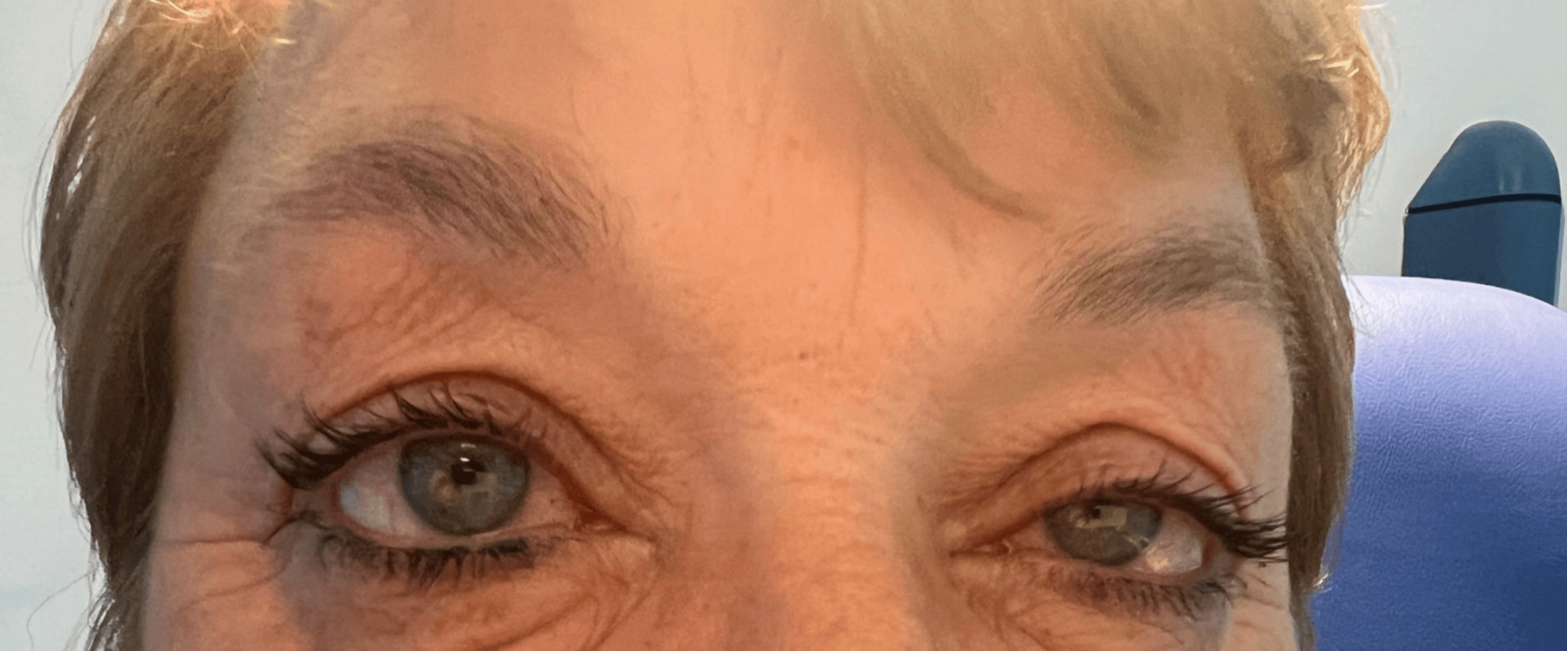
Left-sided partial ptosis with miosis.

Examination revealed signs of left-sided partial Horner syndrome, which included left-sided ptosis and anisocoria (left pupil 2 mm, right pupil 2.5 mm), without evidence of facial anhidrosis (Figure 1). There was mild tenderness over the left maxillary and temporal region, but no signs of facial weakness or sensory deficit. Extraocular movements were full with no ophthalmoplegia, diplopia, or nystagmus. Her cranial nerves were intact, with no motor and sensory deficit detected, and normal gait on neurological examination. 

Blood test results were unremarkable (Table 1). The low CRP level (4 mg/L) makes an active bacterial infection less likely.

**Table 1:** Blood test results.

Test	Result
Hemoglobin (Hb)	148 g/L
White cell count (WCC)	8.9 × 10⁹/L
Platelets	365 × 10⁹/L
C-reactive protein (CRP)	4 mg/L
Prothrombin time (PT)	10.8 seconds
International normalized ratio (INR)	0.9
Renal function	Normal

**Figure 2:**
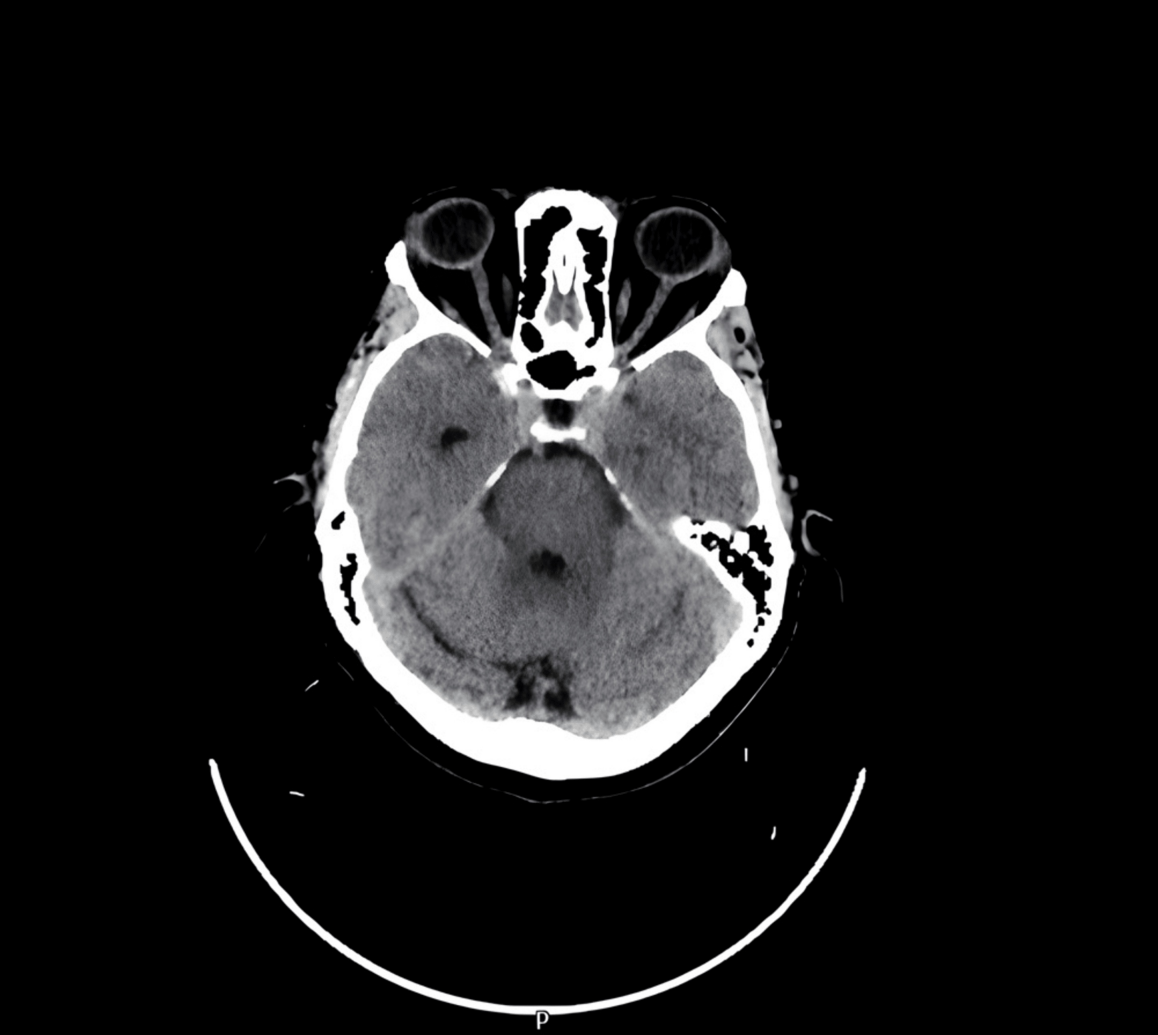
Non-contrast CT head: no acute findings. The petrous bone is intact.

Non-contrast CT head showed no acute intracranial abnormalities, which ruled out intracerebral hemorrhage or mass effect as causes of the patient’s symptoms (Figure 2).

**Figure 3:**
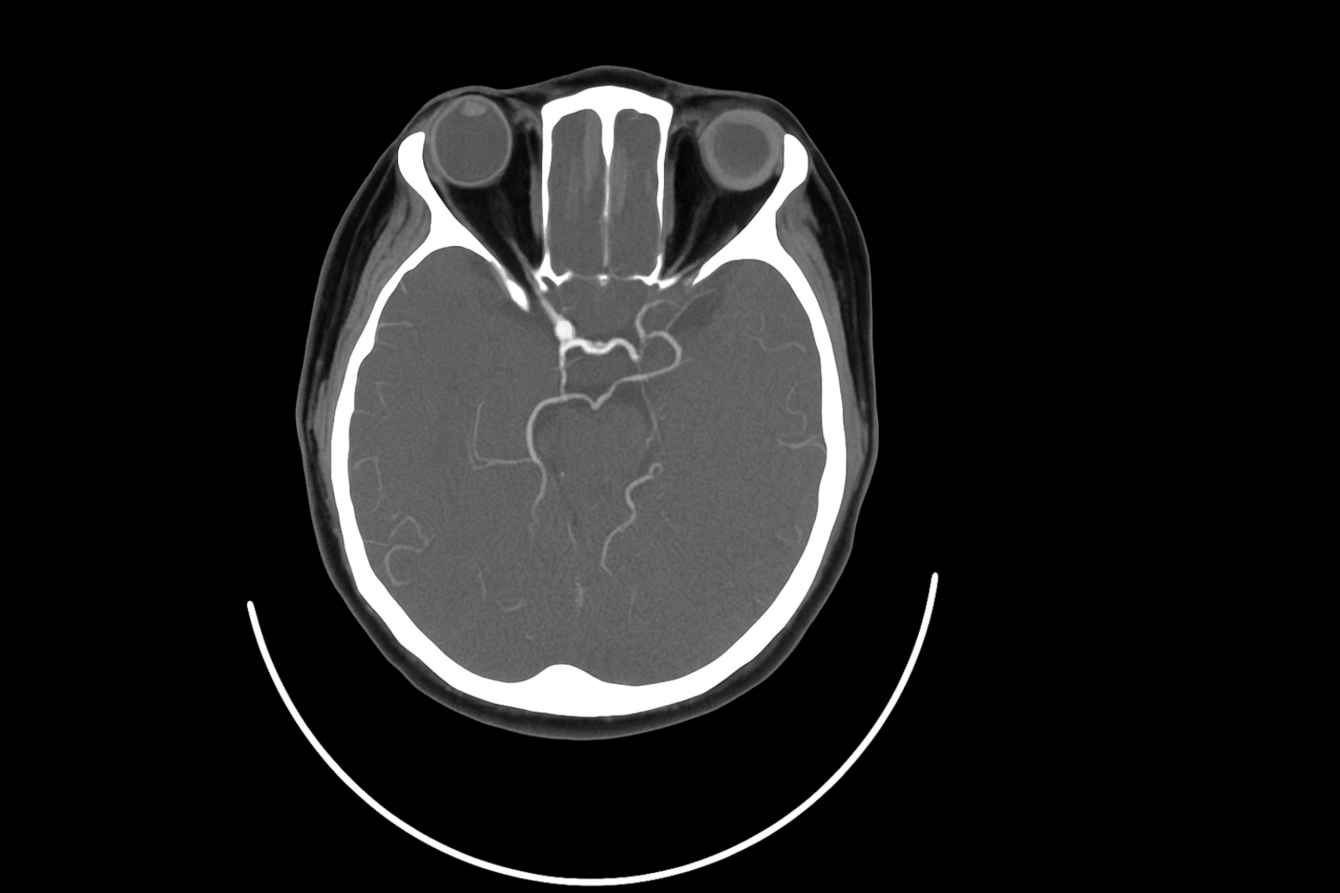
CT angiogram (axial view): normal circle of Willis. No carotid dissection.

Given the presence of painful Horner syndrome, a CT angiogram was performed to rule out carotid artery dissection, cavernous sinus lesion, internal carotid artery aneurysm, or other vascular causes (Figure 3).

**Figure 4:**
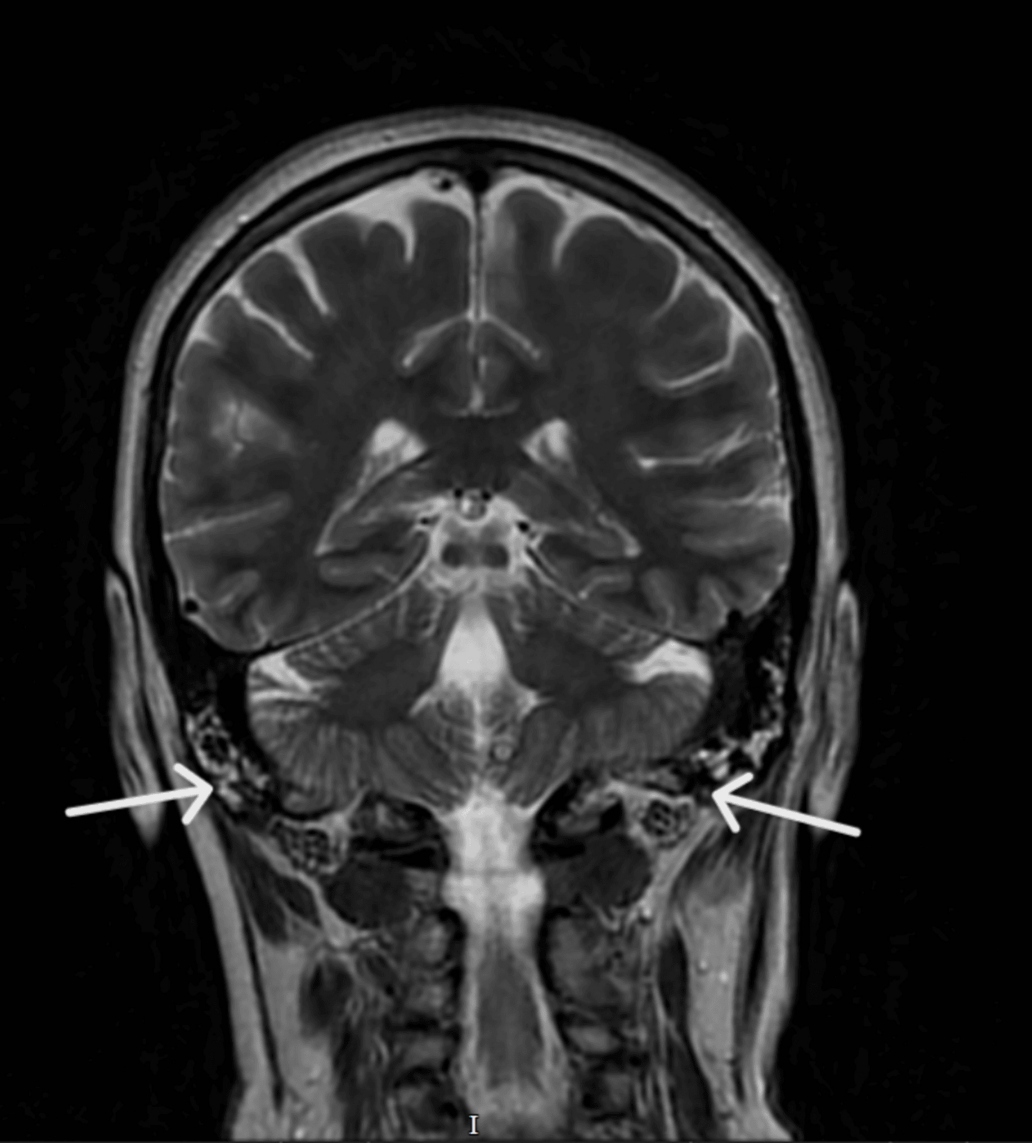
MRI brain (T2 coronal): Bilateral petromastoid inflammation noted, more prominent on the left.

Due to persistent symptoms for a few days after completing the antibiotics and localized facial tenderness, an MRI brain with contrast was arranged, which demonstrated bilateral inflammatory changes in the petromastoid air cells, more pronounced on the left side, consistent with petrous apicitis without evidence of abscess formation or bone erosion (Figure 4) [3]. 

The patient was started on pregabalin 75 mg twice daily for neuropathic pain. At follow-up one week later, she reported marked improvement in facial pain and resolution of ptosis. No new neurological symptoms were noted. She was therefore discharged from SDEC and remains under neurology follow-up, continuing routine botulinum toxin therapy for cervical dystonia (Table 2).

**Table 2:** Clinical timeline of symptom progression and resolution.

Day	Clinical event
Day 0	Onset of left-sided headache and facial pain
Day 3	Development of left eye ptosis
Day 6	Neuroimaging performed
Day 13	Complete resolution of symptoms

## Discussion

Raeder’s paratrigeminal syndrome is an uncommon clinical entity characterized by unilateral facial pain or headache, ipsilateral oculosympathetic paresis (ptosis and miosis), and preserved sweating. It represents a postganglionic variant of Horner syndrome, localizing the lesion to the lesion in the region where the postganglionic sympathetic fibers travel alongside the trigeminal nerve, most notably in the region of the petrous apex and cavernous sinus. Petrous apicitis is a recognized cause, as supported by the MRI findings of petromastoid inflammation in this case.

This patient presented with classic features of painful partial Horner syndrome, initially raising suspicion for serious vascular pathology such as carotid artery dissection - a well-known cause of postganglionic Horner syndrome with associated facial or head pain. CT angiography ruled this out, shifting the focus toward inflammatory or structural causes. Subsequent MRI findings of bilateral petromastoid air cell inflammation marked on the symptomatic side confirmed a diagnosis of petrous apicitis, a rare cause of Raeder’s syndrome.

Petrous apicitis, classically described as part of Gradenigo’s syndrome (which includes abducens nerve palsy and trigeminal neuralgia), can also result in isolated postganglionic Horner if inflammation extends to the lateral petrous apex. Inflammation in this area can affect the postganglionic sympathetic fibers without involving the abducens nerve, leading to a partial Horner syndrome rather than full Gradenigo’s triad. The presence of deep facial pain in the ophthalmic and maxillary divisions of CN V1/V2-along with ipsilateral ptosis and miosis-is a hallmark of this presentation [4].

The absence of anhidrosis in our patient supports the postganglionic localization. Sudomotor fibers responsible for facial sweating typically branch off earlier via the external carotid plexus and would be spared in this anatomical region. The underlying inflammatory process in this case likely originated from subclinical otitis media or sinusitis, which had resolved symptomatically following outpatient antibiotic therapy five weeks earlier. Low-grade petromastoid infections may not always present with overt systemic signs (fever, leukocytosis) or discharge, making imaging critical for diagnosis. 

Given the patient’s history of botulinum toxin injections for spasmodic torticollis, an iatrogenic cause of Horner syndrome was considered. Botulinum toxin injections in the cervical region have been rarely associated with transient Horner syndrome, potentially due to local diffusion affecting the cervical sympathetic chain [5]. Similarly, chiropractic cervical manipulations have been linked to Horner syndrome, likely due to mechanical disruption of the sympathetic pathway [6]. However, the temporal relationship (injections administered every six months, with the last dose given months earlier) and the presence of petromastoid inflammation on MRI made an inflammatory etiology more likely in this case.

Differential diagnoses included carotid dissection, cavernous sinus lesions, and neoplasms, all of which were excluded by CT angiogram and MRI of the brain. Management of Raeder's syndrome is tailored to the underlying etiology. In infectious or inflammatory causes, symptom control with neuropathic agents such as pregabalin or carbamazepine, along with imaging surveillance, may be sufficient if symptoms are improving and there is no suspicion of abscess. In this case, our patient responded favorably to pregabalin, with the resolution of symptoms supporting this approach.

## Conclusions

This case illustrates a rare but clinically important cause of painful partial Horner syndrome, particularly when associated with facial pain and recent upper respiratory or otologic symptoms. The identification of inflammatory changes in the petromastoid region on MRI, in the absence of vascular or neoplastic causes, supports an inflammatory etiology. The anatomical proximity of the petrous apex to the trigeminal ganglion and postganglionic sympathetic fibers explains the patient’s constellation of symptoms.

Consideration of iatrogenic causes, such as botulinum toxin injection or chiropractic manipulations, is warranted in relevant histories, though those were less likely in this case with the given imaging findings.

This case highlights the importance of considering petrous apicitis in patients with painful partial Horner syndrome, even in the absence of overt otologic symptoms or systemic signs.

Timely imaging to exclude life-threatening differentials, such as carotid dissection or cavernous sinus pathology, is critical. Early neuroimaging and neuropathic pain management facilitate symptom resolution, highlighting their therapeutic benefits.
